# Estradiol deficiency as a consequence of aging contributes to the depletion of the satellite cell pool in female mice

**DOI:** 10.1111/acel.14441

**Published:** 2024-12-06

**Authors:** Brian P. Sullivan, Alexie A. Larson, Ahmed S. Shams, Shawna L. McMillin, Mara C. Ebeling, Sydney Peng, Michael Kyba, Dawn A. Lowe

**Affiliations:** ^1^ Division of Physical Therapy and Rehabilitation Science, Department of Family Medicine and Community Health, Medical School University of Minnesota Minneapolis Minnesota USA; ^2^ Lillehei Heart Institute and Department of Pediatrics, Medical School University of Minnesota Minneapolis Minnesota USA; ^3^ Human Anatomy and Embryology Department, Faculty of Medicine Suez Canal University Ismailia Egypt

**Keywords:** aging, estradiol, satellite stem cell, sex hormones, skeletal muscle

## Abstract

The effects of aging on the satellite cell pool have primarily been studied in male mice, where the role of cell‐intrinsic versus environmental changes on satellite cell function remains contentious. Estradiol is necessary for maintenance of satellite cell pool size in adult female mice—here we investigate the hypothesis that in females, estradiol is a major environmental driver of age‐associated effects on satellite cells. In 24–26 month‐old ovarian senescent mice, we find the satellite cell pool size is severely diminished in certain muscles (TA and EDL) but only marginally affected in others (soleus and gastrocnemius). Supplementation with 17‐beta estradiol significantly increases satellite cell pool size in the TA and EDL. To assess cell‐intrinsic versus environmental regulation, we perform two transplantation experiments, Adult or Aged satellite cells transplanted into Adult recipients, and Adult satellite cells transplanted into Adult or Aged mice. These results demonstrate that the aged environment dominates over cell‐autonomous age in terms of the specification of satellite cell pool size. Transcriptional profiling on satellite cells from Adult, Aged and ovariectomized mice revealed commonalities across the two estradiol‐deficient conditions, Aged and ovariectomized, in GO terms from differentially expressed genes. Our findings support the hypothesis that the lack of estradiol contributes to reductions in satellite cell number in Aged female muscle, yet cells that remain are functional in terms of proliferative potential and self‐renewal capacity. These findings have implications for sex hormone treatment of menopausal women and highlight the vital role of estradiol in the maintenance of the satellite cell pool.

## Graphical abstract

In females, estradiol deficiency is a major environmental factor contributing to the age‐associated effects on satellite cells. To assess cell‐intrinsic versus environmental regulation of satellite cells, we perform two transplantation experiments, Adult or Aged satellite cells transplanted into Adult recipients, and Adult satellite cells transplanted into Adult or Aged mice. Our findings demonstrate that the aged, estrogen‐deficient environment dominates over cell‐autonomous age in terms of the specification of satellite cell pool size.

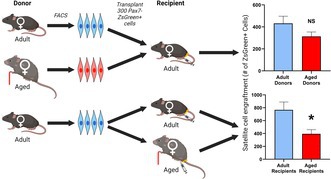

**Abbreviations**
AGC: automated gain control
EDL: extensor digitorum longus
ERa: estrogen receptor alpha
ERb: estrogen receptor Beta
FBS: Fetal Bovine Serum
Gas: gastrocnemius
GPER: G protein coupled estrogen receptor
IS: 1‐methylimidazole‐2‐sulfonyl chloride
MGM: myogenic growth medium
MTBE: tert‐butyl methyl ether
NIA: National Institute on Aging
OVX: Ovariectomy
PI: propidium iodide
Sol: soleus
TA: tibialis anterior

## 1 INTRODUCTION

The maintenance of skeletal muscle mass across the lifespan is vital for physical movement and to support healthspan. Throughout life, skeletal muscle incurs damage and injury, which require regeneration for the maintenance of strength and muscle function (Fry et al., 2015; Larson et al., 2020). Satellite cells are skeletal muscle resident stem cells and are necessary for optimal skeletal muscle regeneration following injury (Fry et al., 2015). Satellite cells reside between the basal lamina and the sarcolemma and are typically quiescent until activated by injury or a growth stimulus (Keefe et al., 2015; Kuang et al., 2007; Mauro, 1961). Upon activation, satellite cells proliferate and divide either symmetrically or asymmetrically with progeny differentiating to fuse and repair damaged or growing muscle fibers or returning to quiescence to maintain the satellite cell pool (Kuang et al., 2007; Pawlikowski et al., 2019; Robinson et al., 2021).

Aging disrupts satellite cell quiescence and the balance between symmetric and asymmetric division (Brack et al., 2007; Chakkalakal et al., 2012; Sousa‐Victor et al., 2014; Tierney et al., 2018), depleting and impairing the satellite cell pool (Keefe et al., 2015; Sajko et al., 2004; Verdijk et al., 2014) in certain locomotory muscles of male mice, but not in other locomotory muscles, diaphragm, or masseter of male mice (Arpke et al., 2021). Whether through declines in number or declines in function of satellite cells, the regenerative potential in skeletal muscle of aged males is significantly reduced compared to adult males (Brack et al., 2005; Chakkalakal et al., 2012; Keefe et al., 2015; Shefer et al., 2006). Evidence exists for both alterations in cell intrinsic factors as well as in the environment, including circulating factors (i.e., hormones), contributing to disruptions in quiescence in male aged muscle (Brack & Rando, 2007; Dumont et al., 2015). The loss of satellite cells with aging in females is less studied with some indication that female muscle may have a more substantial decline in satellite cell number with age than males (Day et al., 2010). Whether female age‐associated deficits are due to cell‐intrinsic factors or alterations in the satellite cell environment is unclear.

Of particular relevance to aged female muscle is the sex hormone, 17β estradiol, due to mounting evidence that estradiol influences satellite cell maintenance and muscle regeneration in adults (Collins et al., 2019; Larson et al., 2020, 2022). Estradiol is the primary sex hormone in females and serum levels fall drastically at the perimenopausal transition in women (Messier et al., 2011) and after ovarian senescence in rodents (Gilmer et al., 2023). Estradiol deficiency reduces skeletal muscle mass and strength in females and limits functional recovery following repeated injuries in rodents (Greeves et al., 1999; Larson et al., 2020; Phillips et al., 1993). Recently, we have demonstrated that estradiol is a potent regulator of satellite cells in adult female mice and that deficiency compromises the ability of satellite cells to self‐renew and differentiate into muscle fibers (Collins et al., 2019). Utilizing a combination of ovariectomy (Ovx; bilateral removal of the ovaries) and hormone replacement, we have demonstrated that estradiol is important for the maintenance of satellite cells in female mice (Collins et al., 2019). Estradiol acts through three primary receptors: estrogen receptor α (ERα), estrogen receptor β (ERβ), and G protein‐coupled estrogen receptor (GPER). Estradiol primarily signals through ERα in skeletal muscle and signaling through this receptor is necessary to prevent apoptosis in satellite cells (Collins et al., 2019; Kamanga‐Sollo et al., 2013; Velders et al., 2012). To date, the majority of aging research on skeletal muscle regeneration has been conducted in males and minimal research has been performed on aging females; consequently, robust recapitulation of aging's effects on skeletal muscle and satellite cells have not been demonstrated in females.

Here we investigated the hypothesis that the loss of estradiol with aging in female mice contributes to the depletion of the satellite cell pool. To test this hypothesis, we supplemented ovarian senescent female mice with estradiol or placebo and assessed satellite cell number. Additionally, we performed transplantations, wherein we isolated satellite cells from Adult or Aged mice and transplanted them into injured muscles of Adult recipient female mice and measured their ability to engraft and self‐renew. We also performed the reciprocal experiment, namely comparing the size of the satellite cell pool reconstituted by Adult cells in Adult versus Aged mice. Satellite cell cycle and colony‐forming analyses were also conducted on muscle from Adult and Aged female mice to assess effects of aging on satellite cell function. Finally, we evaluated transcriptional profiles of satellite cells from Adult, Aged and Adult Ovx female mice to identify similarities in the transcription patterns of Aged and Ovx mice, two conditions involving the loss of estradiol.

## 2 METHODS

### 2.1 Experimental design and mice

All procedures were performed in accordance with protocols approved by the Institutional Animal Care and Use Committees at the University of Minnesota (#A3456‐01). All experiments were conducted on female mice when they were Adults (3–5 months of age) or Aged (26–28 months). Female wildtype (C57BL/6) mice were obtained from the National Institute on Aging (NIA). Female Pax7‐ZsGreen mice were generated in‐house (40). All mice were housed in groups of 4–5 and had access to phytoestrogen‐free rodent chow (Harlan‐Tekland #2019; Indianapolis, IN, USA) and water ad libitum. The housing room was maintained on a 14:10 light: dark cycle with controlled temperature at 25°C and humidity.

#### 2.1.1 Experiment 1

To characterize the effects of aging on skeletal muscle and satellite cell number of female mice, we quantified the number of satellite cells from individual but entire hindlimb muscles (tibialis anterior [TA], extensor digitorum longus [EDL], soleus [Sol] and gastrocnemius [Gas]) of Adult and Aged Pax7‐ZsGreen female mice by FACS (up to *n* = 11/group). Whole blood was collected via cardiac puncture for the measurement of serum estradiol in a subset of mice (*n* = 6/group).

#### 2.1.2 Experiment 2

The onset of ovarian senescence was identified between the age of 20–24 months. This was done by tracking the estrous cycle of mice for 3–5 consecutive days via vaginal cytology to confirm normal estrous cycles or persistent diestrus, the latter being evidence of ovarian senescence. Ovarian senescent mice were then randomized to receive either a placebo or 17β‐estradiol (0.09 mg/60 days) pellet. Eight weeks later, a second pellet of the same type was implanted and mice were euthanized 8 weeks later (16 weeks of treatment) (*n* = 5/group). Flow cytometry was performed to quantify satellite cell number in individual hindlimb muscles. During the 16‐week period, 2 mice in the estradiol treatment group were euthanized due to rapidly declining physical health.

#### 2.1.3 Experiment 3

To study whether intrinsic alterations in satellite cell function occurs during aging in female muscle, satellite cells from both Adult and Aged Pax7‐ZsGreen female mice (*n* = 5/group) were isolated via FACS and transplanted into irradiated and injured TAs of Adult C57BL/6 female mice (*n* = 15 mice (30 TAs)/group). The isolated, Pax7‐ZsGreen+ cells were either transplanted into the recipient animals, analyzed for cell cycle stage or proliferation and self‐renewal via colony‐forming assay. Engraftment of ZsGreen+ cells was measured 28 days posttransplantation via FACS. Additionally, a second transplantation experiment was performed, wherein satellite cells isolated from Adult Pax7‐ZsGreen female mice (*n* = 4) were isolated via FACS and transplanted into irradiated and injured TAs of Adult (*n* = 10) and Aged (*n* = 11) C57BL/6 female mice. Engraftment of ZsGreen+ cells was measured 28 days posttransplantation via FACS and sorted to test EdU incorporation in the first 30 h posttransplantation or proliferation and self‐renewal via colony‐forming assay. One mouse from each recipient group was euthanized early due to deteriorating health and are excluded from the results.

#### 2.1.4 Experiment 4

To analyze the extent to which the loss of estradiol alters satellite cell gene expression, we performed RNASeq analysis on satellite cells isolated from bulk hind limb muscles of 3 groups of female Pax7‐ZsGreen mice; (1) Adult (4 month) ovary intact, (2) Adult (4 month) ovariectomized (Ovx) and (3) Aged (26 month), ovary intact mice (*n* = 4/group). 14 days after Ovx mice were euthanized and satellite cells isolated for RNA‐Seq analysis. Aged versus Adult and Ovx versus Adult ovary intact differentially expressed gene lists were generated for comparisons between groups. A subset of mice from this group were combined with mice from experiments 1 and 3 to analyze the relationship between body mass and hindlimb muscle mass.

### 2.2 Surgical procedures

Under aseptic conditions, bilateral ovariectomy was performed through two small dorsal incisions between the iliac crest and the lower ribs as previously described (Moran et al., 2007). Pellet implantations were performed as previously described (Larson et al., 2022) on Aged mice following confirmation of ovarian senescence by vaginal cytology. Briefly, mice were given a subcutaneous injection of slow‐release buprenorphine (1 mg/kg) and 2–4 h later were anesthetized by inhalation of isoflurane (2–3%, 125 mL O2 per min). Mice were implanted with placebo pellets or pellets containing 0.09 mg 17β‐estradiol released over a 60 days period (Innovative Research of America, Sarasota, FL). A second pellet was implanted at 60 days. Mice were monitored daily for 3 days following surgery.

### 2.3 Serum estradiol measurement/mass spectrometry

Whole blood was obtained via cardiac puncture and serum isolated from whole blood. Whole blood samples were allowed to clot for 90 min at room temperature. Clot adhesion was disrupted by running a pipette tip around the tube wall prior to centrifugation of samples at 2000*g* for 15 min at room temperature. 200 μL of serum was pipetted to a new microcentrifuge tube and spiked with 100 pg/mL 13C6‐labeled E2 followed by liquid–liquid extraction using tert‐butyl methyl ether (MTBE). Layer partition was achieved by centrifugation (10 min, 3000 g at 4°C), and freezing at −80°C for 30 min. The upper organic layer was transferred to a new vial, and dried down under N2. Samples were derivatized using 1‐methylimidazole‐2‐sulfonyl chloride (IS) as described previously (Li & Franke, 2014).

The analysis of the IS derivative of E2 was carried out on an Orbitrap Lumos Tribrid Mass Spectrometer coupled with an UltiMate 3000 RSLCnano UPLC system (Thermo Scientific, Waltham, MA). Chromatographic separation was obtained on a Phenomenex Luna C8 column (150 × 2 mm, 5 μm, 100 Å) using a mobile phase consisting of 0.1% formic acid in water (A) and acetonitrile (B). Elution was achieved by holding at 30% B for 2 min followed by a linear gradient to 60% B in 11 min, then to 70% B in 2 min with a 1 min hold before increasing B to 95% in 1 min. Column equilibration was performed at initial conditions for 4 min prior to the next injection. The flowrate was 200 μL/min, with a decrease to 150 μL/min during analyte elution (15–17 min).

MS analysis was performed in positive ion targeted selected ion monitoring mode (1.5 amu isolation window) at 120,000 resolution. The mass spectrometer was operated with an electrospray source and an ion transfer tube temperature of 350°C, spray voltage of 4.5 kV, and sheath gas and aux gas values of 30 and 5 units, respectively. The in‐source collision induced dissociation energy was 5 eV and the RF lens value was 70%. The normalized automated gain control (AGC) target was 1000% and an extracted ion signal within 5 ppm of theoretical masses 417.18426 and 423.20439 for the analyte and the 13C6‐labeled internal standard, respectively, was used for quantitation.

### 2.4 Satellite cell isolation

Isolation of satellite cells from bulk hind limb or whole individual muscles (e.g., TA, EDL, Sol, and GC) were performed as described in detail previously (Arpke et al., 2021; Collins et al., 2019). Briefly, muscles were dissected, minced in parallel with muscle fibers, and digested with collagenase type II and dispase (17101–015 and 17,105–041, respectively; Gibco, Grand Island, NY). For isolation of satellite cells from C57BL/6 female mice, mononuclear cells were stained using an antibody mixture of 1 μL PE‐Cy7 rat anti‐mouse CD31 (clone 390; 561,410; BD Biosciences, San Diego, CA), 1 μL PE‐Cy7 rat anti‐mouse CD45 (clone 30‐F11; 552,848; BD Biosciences), 1 μL Biotin rat anti‐mouse CD106 (clone 429 (MVCAM.A); 553,331; BD Biosciences), 1 μL PE Streptavidin (554,061; BD Biosciences), and 2 μL alpha7 integrin 647 (clone R2F2; AbLab; Vancouver, B.C., Canada). Samples were incubated with antibody cocktail, washed, and resuspended with FACS staining medium (2% Fetal Bovine Serum [FBS; 16,000,044; Gibco] in phosphate‐buffered saline [PBS]) containing 0.5 μg/mL propidium iodide (PI) for analysis on a FACSAriaII SORP (BD Biosciences, San Diego, CA). Total satellite cells (lineage negative; VCAM, alpha7 double‐positive cells) were analyzed from the entire muscle sample.

To obtain satellite cells from Pax7‐ZsGreen mice, mononuclear cells were isolated as described above and were incubated in FACS staining medium containing PI and ZsGreen+ (Arpke et al., 2013). Absolute satellite cell counts by FACS were confirmed through gating ZsGreen+ cells and counting beads (CountBright absolute counting beads; C36950; Lot No. 2361079; Invitrogen, Waltham, MA) according to the manufacturer's instructions. Researchers were blinded during all FACS analysis.

### 2.5 Transplantation assay

Transplant recipient mice were anesthetized with intraperitoneal injections of 150 mg/kg ketamine plus 10 mg/kg xylazine and both hind limbs were subjected to a 900 cGy dose of irradiation using an RS 2000 Biological Research Irradiator (Rad Source Technologies, Inc., Suwanee, GA). Lead shields limited exposure to the hind limbs only. 24 h following irradiation, 15 μL of cardiotoxin (10 mM in PBS, Sigma‐Aldrich, Saint Louis, MO) was injected into both TA muscles of each mouse with a Hamilton syringe. 24 h following cardiotoxin injection, 300 ZsGreen+ cells were resuspended into 10 μL of sterile saline and injected into each TA. Both TA's were harvested 28 days' post‐transplantation and prepared for FACS analysis as described above.

### 2.6 Colony‐forming assay

The satellite cell colony‐forming assay was performed as described in detail previously (Shams & Kyba, 2023). Single Pax7‐ZsGreen+ cells isolated by FACS were sorted directly into 0.1% gelatin‐coated 96‐well plates containing myogenic growth medium (MGM): Dulbecco's modified Eagle's medium/F12 without phenol red containing 4.00 mM L‐glutamine, 4.5 mg/mL glucose, sodium pyruvate, 20% Charcoal Stripped‐FBS, 10 ng/mL human basic fibroblast growth factor (bFGF; 100‐ 18C; Peprotech), 1% Pen/Strep, and 1% Glutamax. After culturing plates for 8 days at 37°C and 5% CO_2_, cells were fixed with 4% PFA for 20 min at room temperature. For immunostaining of colonies, cells were permeabilized with 0.3% Triton‐X100 for 20 min at room temperature, washed with PBS, and blocked with 3% BSA in PBS for 1 h at room temperature. Colonies were stained with MF‐20 antibody supernatant (Developmental Studies Hybridoma Bank, University of Iowa; 1:20 dilution) in 3% BSA in PBS overnight at 4°C. After PBS washes, cells were incubated with Alexa Fluor 555 goat anti‐mouse secondary antibody (Life Technologies; 1:500 dilution) in the dark for 45 min at room temperature. The cells were then incubated in the dark with DAPI (300 nM) in PBS for 20 min at room temperature. Colonies were imaged at 10× magnification, on a Zeiss Observer.Z1 inverted microscope equipped with an AxioCam MRm camera (Thornwood, NY). Intensity thresholding in ImageJ was used to measure the number of nuclei and number of colonies by blinded assessors. The cloning efficiency was calculated by dividing the number of colonies in each plate by the number of wells (94 wells per mouse) in which a single cell was sorted then multiplying by 100.

### 2.7 Proliferation assay

Pax7‐ZsGreen+ satellite cells were sorted into MGM and plated onto 0.1% gelatin‐coated 96‐well plates. After allowing satellite cells to adhere for 22 h, cultures were treated with 10 μM of EdU for 8 h. Cells were washed with PBS and fixed with 4% PFA for 20 min at room temperature. Wells were then washed and incubated in 100 mm Tris–HCl, pH 8.5 (Boston Biosciences, Royal Oak, MI, USA), 1 mm CuSO_4_ (Acros; ThermoFisher Scientific), 2.5 mm TAMRA Azide 568 (Invitrogen) and 100 mm ascorbic acid (ThermoFisher Scientific) for 30 min. Prior to imaging, nuclei were costained with DAPI. Myoblasts were imaged immediately via fluorescence microscopy for colocalization of EdU to DAPI. The percentage of proliferating cells was determined using ImageJ (National Institutes of Health, Bethesda, MD, USA).

### 2.8 Cell cycle analysis

Pax7‐ZsGreen+ satellite cells isolated by FACS were fixed by adding cooled 100% EtOH dropwise while vortexing the cell suspension in PBS to a final concentration of 70% EtOH. Cells were allowed to fix overnight at −20°C then centrifuged at 500 *g* for 5 min, then resuspended and washed with PBS. Cells were incubated in a staining solution containing 0.1% (vol/vol) Triton‐X100 in PBS, 2 mg DNase‐free RNase (Sigma), and 1 mg/mL PI for 30 min at RT. Samples were analyzed on a FACSAriaII SORP (BD Biosciences, San Diego, CA). Cell cycle distributions for satellite cells in G1, S, and G2 phases were performed using FlowJo v.10 univariate modeling with the Watson pragmatic algorithm by blinded assessors.

### 2.9 RNA sequencing

Pax7‐ZsGreen+ satellite cells were isolated from bulk muscles of Adult, Ovx, and Aged female mice by FACS and given to the University of Minnesota Genomics core. Total RNA was isolated using the RNeasy Plus Universal Mini Kit from Qiagen according to manufactures instructions with one modification. In place of gDNA elimination solution, the hstep was split with RWT buffer and on‐column DNase digestion. Total eukaryotic RNA isolates were quantified using a fluorimetric RiboGreen assay. Total RNA integrity was assessed using capillary electrophoresis (e.g., Agilent BioAnalyzer 2100), generating an RNA Integrity Number (RIN). For samples to pass the initial QC step, SMARTer Stranded Pico Mammalian kit recommends at least 250 pg. total RNA and RIN values of higher than 2–3. All samples had ≥2.27 ng of total RNA and RIN values ≥7.5. Samples were then converted to Illumina sequencing libraries.

Total RNA samples were converted to Takara sequencing libraries using Takara Bio's SMARTer Stranded Total RNA‐Seq—Pico Mammalian Kit v2 (Cat. # 634414). Please see www.takarabio.com for a detailed list of kit contents and methods. In summary, between 250 pg.—10 ng of total RNA is fragmented (fragmentation is skipped if samples are sufficiently degraded) and then reverse transcribed into cDNA using random primers. The Template Switching Oligo (TSO) was incorporated during cDNA synthesis and allows for full length cDNA synthesis and strand specificity to be retained. Illumina sequencing adapters and barcodes were then added to the cDNA via limited PCR amplification. Next, mammalian ribosomal cDNA was enzymatically cleaved. Uncleaved fragments were PCR‐enriched 12–16 cycles. The final library size distribution was validated using capillary electrophoresis and quantified using fluorimetry (PicoGreen). Indexed libraries are then normalized and pooled for sequencing.

Pooled libraries were denatured and diluted to the appropriate clustering concentration. The libraries were then loaded onto the NovaSeq‐paired end flow cell and clustering occurs on board the instrument. Once clustering was complete, the sequencing reaction immediately begins using Illumina's 2‐color SBS chemistry and 2 separate 8 or 10 base pair index reads are performed. Finally, the clustered library fragments are re‐synthesized in the reverse direction thus producing the template for paired end read 2.

Base call (.bcl) files for each cycle of sequencing were generated by Illumina Real Time Analysis (RTA) software. The base call files and run folders were streamed to servers maintained at the Minnesota Supercomputing Institute. Primary analysis and de‐multiplexing were performed using Illumina's bcl2fastq v2.20. 2 × 50 bp FastQ paired end reads for 18 samples (*n* = 45.9 million average reads per sample) were trimmed using Trimmomatic (v 0.33) enabled with the optional, “headcrop −3” option, “−q” option; 3 bp sliding‐window trimming from 3′ end requiring minimum Q30.

Quality control on raw sequence data for each sample were performed with FastQC. Read mapping was performed via Hisat2 (v2.1.0) using the mouse genome (Mus musculus GRCm38) as reference. Gene quantification was done via Feature Counts for raw read counts. Differentially expressed genes were identified using the edgeR (negative binomial) feature in CLCGWB (Qiagen, Redwood City, CA) using raw read counts.

Genes were included in the differential expression analysis if they had greater than 0 reads in at least 1 sample per group. RNA transcripts were considered significant and differentially expressed if they had an adjusted *p*‐value of ≤0.05 and were defined as upregulated if they had a Log_2_Fold change ≥0.5 or downregulated if they had a Log_2_Fold change ≤ −0.5. Volcano plots were created using the EnhancedVolcano package in RStudio (version 2023.09.1; Build 494).

### 2.10 Gene ontology (GO) enrichment analysis

Differentially expressed genes for (1) Aged versus Adult and (2) Ovx versus Adult (ovary intact) were used for GO enrichment analysis using the compareCluster function in the clusterProfiler package (Version 4.9.5) in RStudio. Comparative enrichment analysis was performed for GO terms in all three domains; biological processes, molecular functions and cellular components. GO annotation terms were considered significant if they had a Benjamini – Hochberg adjusted *p*‐value of ≤0.05. Comparative dot plots were created using EnrichPlot in RStudio.

### 2.11 Statistical analyses

All data were analyzed via unpaired students t tests for determining significant differences between Adult and Aged groups. Significance was established at *α* < 0.05. RNASeq data was analyzed and visualized in RStudio (Version 4.3.1) and figures arranged in BioRender. All non‐RNASeq data were graphed and analyzed in GraphPad Prism (Version 8.2.1).

## 3 RESULTS

### 3.1 Phenotypes of aged female mice

**FIGURE 1:**
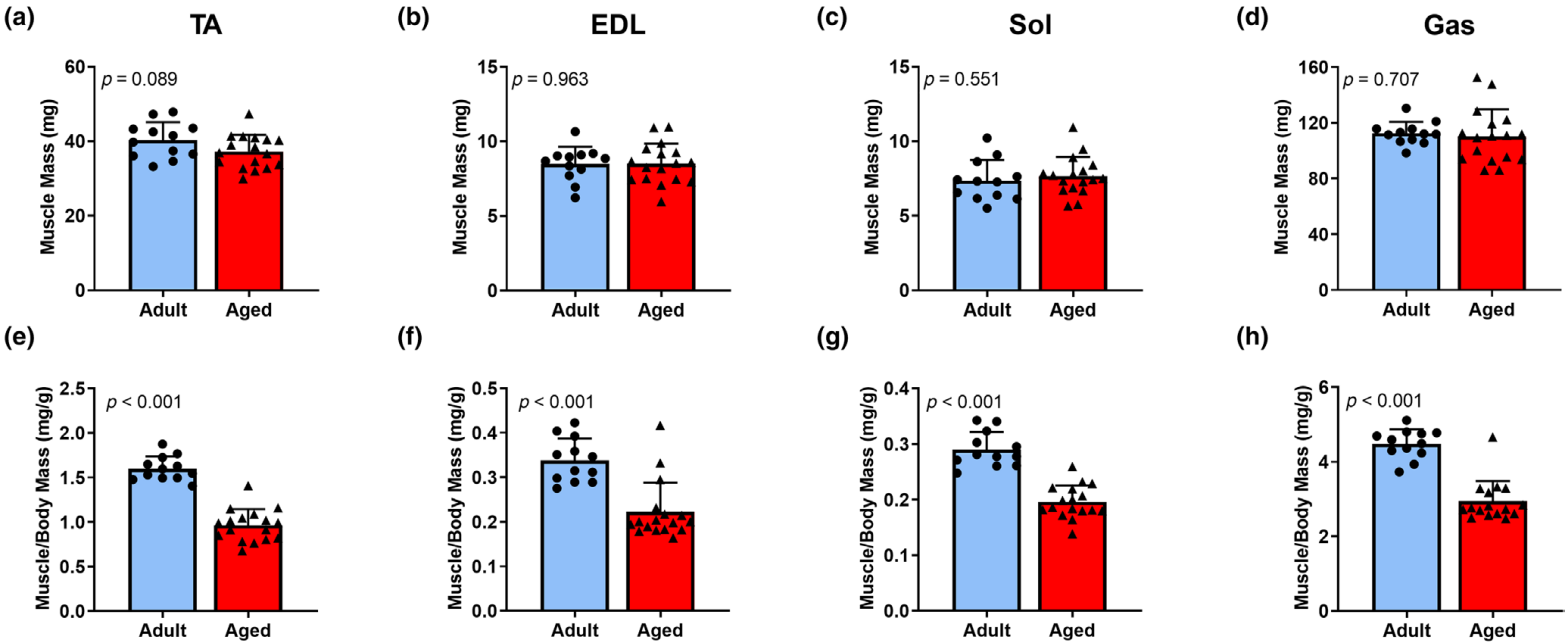
Aging alters the relationship between body mass and muscle mass in female mice. Muscle masses of the tibialis anterior (TA) (a), extensor digitorum longus (EDL) (b), soleus (Sol) (c), and gastrocnemius (Gas) (d) muscles, and the ratio of those muscles to body mass (e‐h) in Adult and Aged female mice. Mean ± SD. Data are from mice utilized in Experiments 1, 3 and 4. *n* = 12 adult/17 aged.

**FIGURE 2:**
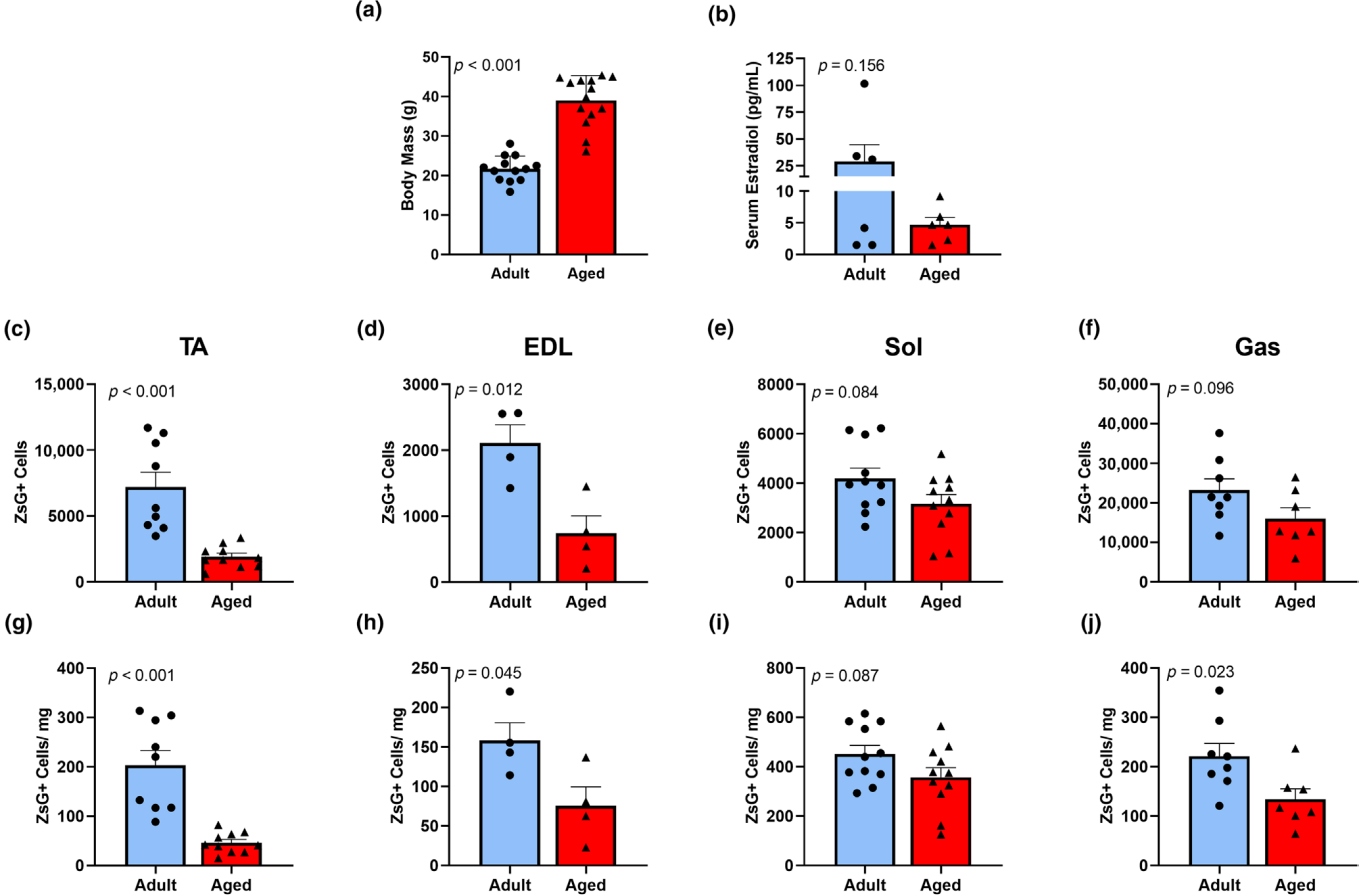
Body and uterine masses are greater and satellite cell number is lower in hind limb muscles of Aged versus Adult mice. Total body mass (a) and serum estradiol (b) in Adult and Aged female mice. Total number of Pax7ZsGreen + cells in the tibialis anterior (TA) (c), extensor digitorum longus (EDL) (d), soleus (Sol) (e) and gastrocnemius (Gas) (f) and the number of ZsGreen+ cells /mg of muscle mass in those four muscles (g–j) of Adult and Aged female mice. These data are from mice used in experiment 1, 3 and 4. Mean ± SD.

While aging is associated with a loss of skeletal muscle mass and strength in males, relatively little research has characterized aging in female mice. We evaluated absolute muscle masses of 4 hind limb muscles, the TA, EDL, Sol, and Gas from 12 Adult and 17 Aged female mice and observed no significant differences between Adult and Aged mice (*p* ≥ 0.089; Figure 1a–d). However, the relationship between muscle mass and body mass was significantly altered (*p* < 0.001; Figure 1e–h). Aged mice had significantly higher body masses than Adult mice (*p* = 0.022; Figure 2a). Serum estradiol was low in Aged mice but not significantly different from Adult mice (*p* = 0.156; Figure 2b) due to the expected variability over the estrous cycle in adults. Absolute Pax7‐ZsGreen+ satellite cell numbers in TA and EDL muscles were ~ 74% and 65% lower, respectively, in Aged versus Adult mice (*p* ≤ 0.012; Figure 2c,d). Absolute satellite cell numbers were not significantly different between Aged and Adult in Sol and Gas muscles (*p* ≥ 0.084; Figure 2e,f). The number of Pax7‐ZsGreen+ satellite cells per mg of muscle was 40–78% lower in 3 of the 4 hindlimb muscles of Aged versus Adult mice (*p* ≤ 0.045; Figure 2g,h,j) but failed to reach significance in the Sol muscle (*p* = 0.087; Figure 2i).

### 3.2 Estradiol partially rescues the loss of satellite cells in aged female mice

**FIGURE 3:**
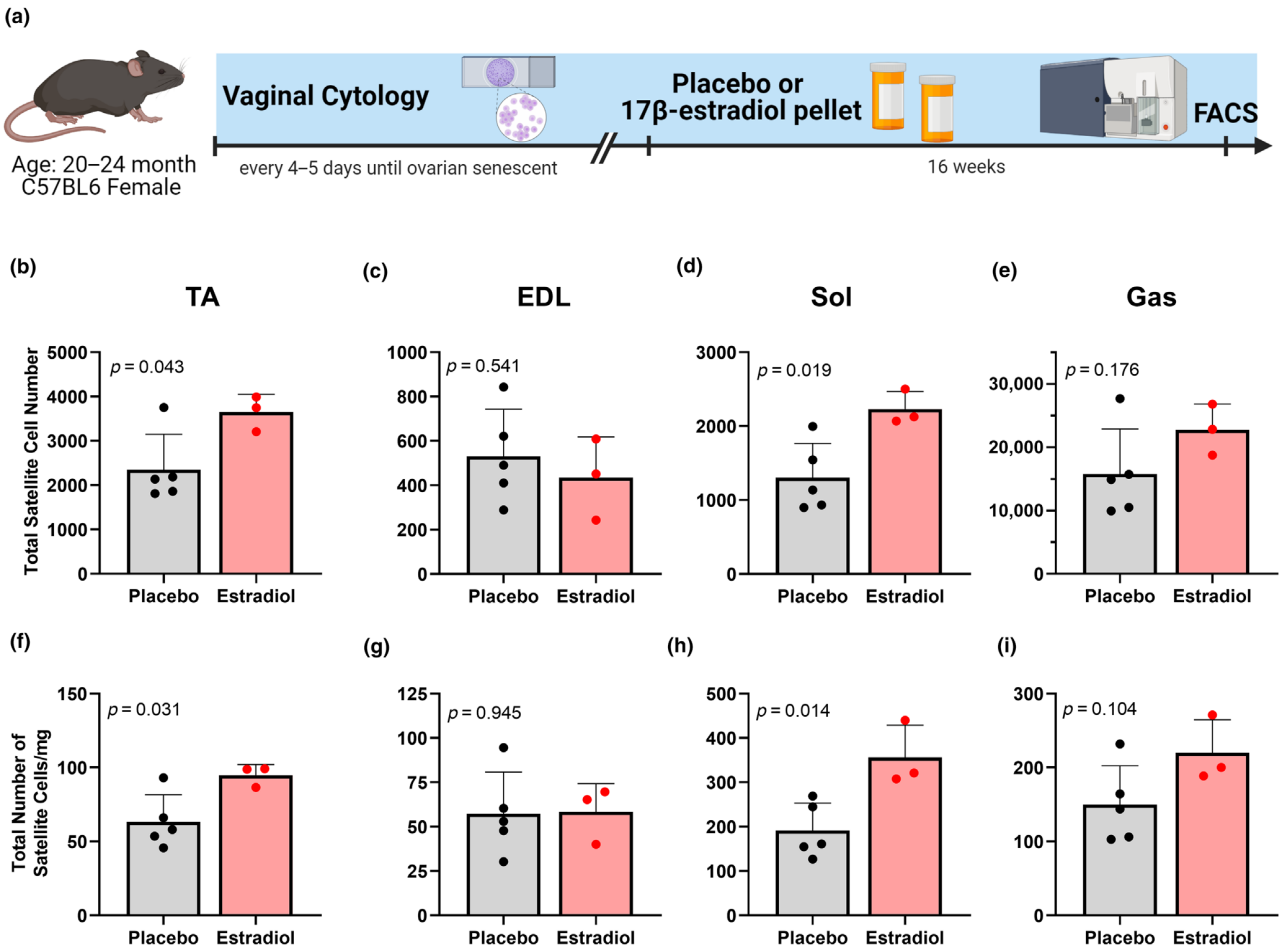
Ovarian senescent mice that received 17β‐estradiol treatment have higher satellite cell number than placebo‐treated mice. Experimental timeline of mice treated with Placebo or Estradiol (a). The total number of satellite cells in TA (b), EDL (c), soleus (Sol) (d) and gastrocnemius (Gas) (e) muscles and the total number of satellite cells per mg of muscle mass in the same muscles (f–i) from ovarian senescent female mice. Mean ± SD.

To directly assess whether estradiol is an ovarian hormone contributing to the loss of satellite cell number in Aged female muscle, 24‐month, ovarian senescent Pax7‐ZsGreen mice were treated with placebo or estradiol via pellet implantation for 16 weeks. Mice that received estradiol had a greater number of satellite cells in TA and Sol muscles compared to mice that received placebo whether expressed as the absolute number of satellite cells (*p* ≤ 0.043; Figure 3b,d) or as satellite cells relative to muscle mass (*p* ≤ 0.031; Figure 3f,h). Gas muscles from estradiol‐treated mice trended toward having more satellite cells than placebo‐treated (*p* ≥ 0.104; Figure 3e,i) while EDL muscles did not differ between estradiol and placebo groups (*p* ≥ 0.541; Figure 3c,g).

### 3.3 Cell intrinsic factors do not alter satellite cell function in aged females

**FIGURE 4:**
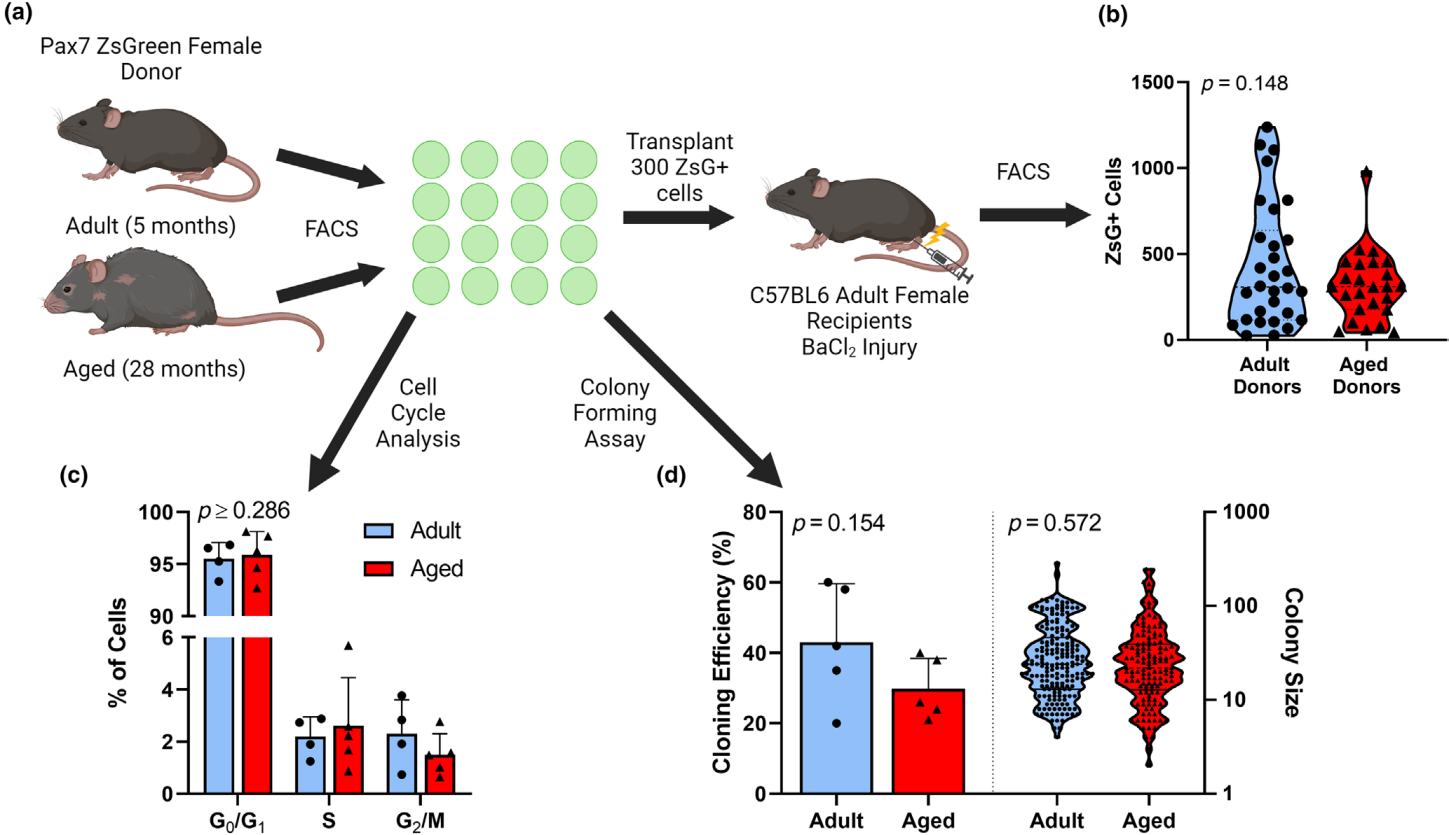
Satellite cells from Adult and Aged female mice have similar muscle engraftment in an Adult, estradiol replete environment and show similar self‐renewal and proliferative capacities in vitro compared to an Aged, estradiol deficient environment. Experimental timeline (a), engraftment of ZsGreen+ cells into TA muscle of recipient mice (b), cell cycle stage of transplanted cells (c), and clonal ability (d) from Adult and Aged donors. Mean ± SD.

To further probe the extent that environmental and cell intrinsic factors influence self‐renewal of satellite cells, 300 Pax7‐ZsGreen+ satellite cells from Adult (estradiol replete) and Aged (estradiol deficient) Pax7‐ZsGreen female mice were transplanted into previously irradiated, cardiotoxin injured TA muscles of Adult, recipient C57BL/6 female mice (Figure 4a). Age of donor had no effect on the ability of satellite cells to engraft into the recipient animals (*p* = 0.148, Figure 4b). From the same two groups of donor mice, clonogenicity of satellite cells was also assessed. The ability of satellite cells from Adult and Aged mice to survive and form colonies in the same in vitro environment did not differ as measured by cloning efficiency (*p* = 0.154) and colony size (Figure 4c) (*p* = 0.572). Additionally, cell cycle analysis revealed no difference in cell cycle stage of Adult versus Aged satellite cells (Figure 4d) further indicating that the satellite cell environment plays a larger role on satellite cell proliferation and self‐renewal than cell intrinsic factors.

### 3.4 The aged environment significantly alters satellite cell function

**FIGURE 5:**
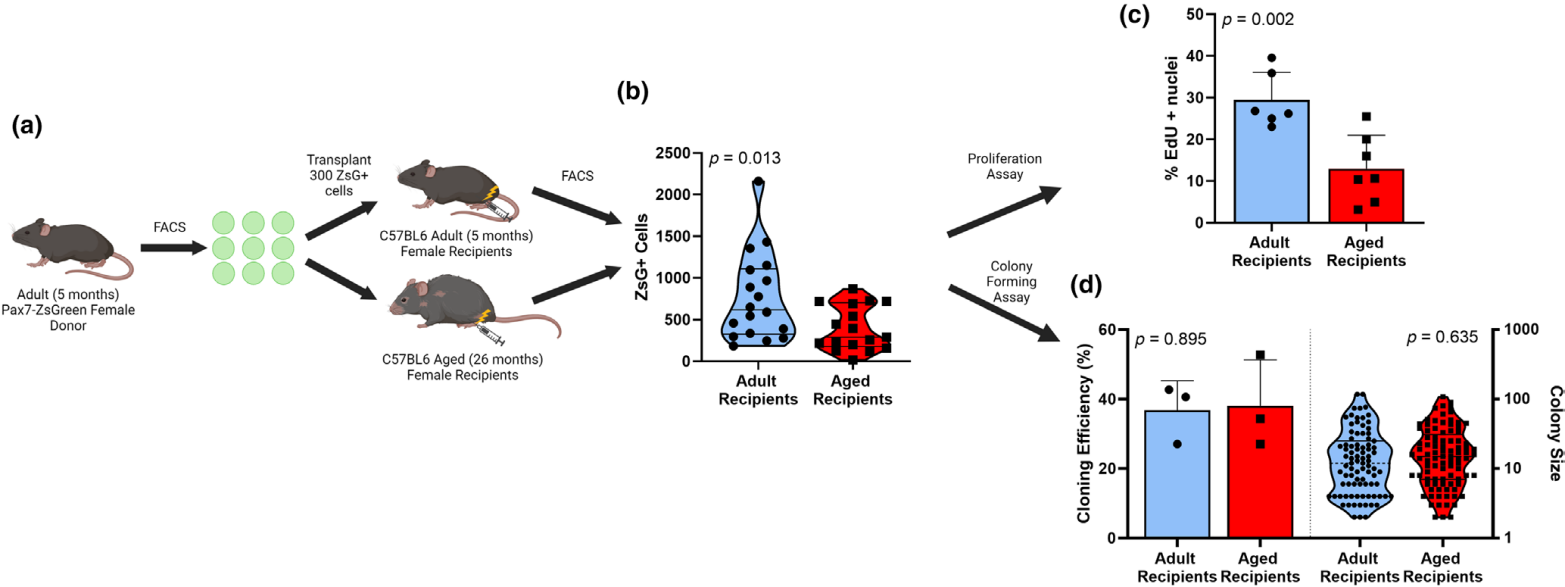
Satellite cells from Adult female mice show altered behavior when transplanted into an Aged versus an Adult environment. Experimental timeline (a), engraftment of ZsGreen+ cells into TA muscle of recipient mice (b), EdU incorporation in the first 30 h following transplant (c), and clonal ability (d) of transplanted satellite cells reisolated from Adult and Aged recipient mice. Mean ± SD.

To assess environmental effects independent of age of cells, we transplanted 300 Pax7‐ZsGreen+ satellite cells from Adult Pax7‐ZsGreen female mice into previously irradiated, cardiotoxin injured TA muscles of Adult (estradiol replete) or Aged (estradiol deficient) recipient C57BL/6 female mice and evaluated the size of the donor‐derived satellite cell pool 28 days later (Figure 5a). The same grafts reconstituted significantly smaller satellite cell pools in Aged recipients compared to Adult recipients (*p* = 0.013; Figure 5b). During sorting, a portion of the Pax7‐ZsGreen+ satellite cells isolated from Adult and Aged recipients were cultured and EdU incorporation and clonogenicity were assessed. EdU incorporation was reduced in Pax7‐ZsGreen+ satellite cells following 28 days in an aged environment compared to those transplanted into an adult environment (*p* = 0.002; Figure 5c). However, the ability of Adult Pax7‐ZsGreen satellite cells isolated from Adult and Aged recipient mice to survive and form colonies in vitro did not differ as measured by cloning efficiency (*p* = 0.895; Figure 5d) and colony size (*p* = 0.635; Figure 5d).

### 3.5 Estradiol is a primary environmental factor that is lost with aging in female mice

**FIGURE 6:**
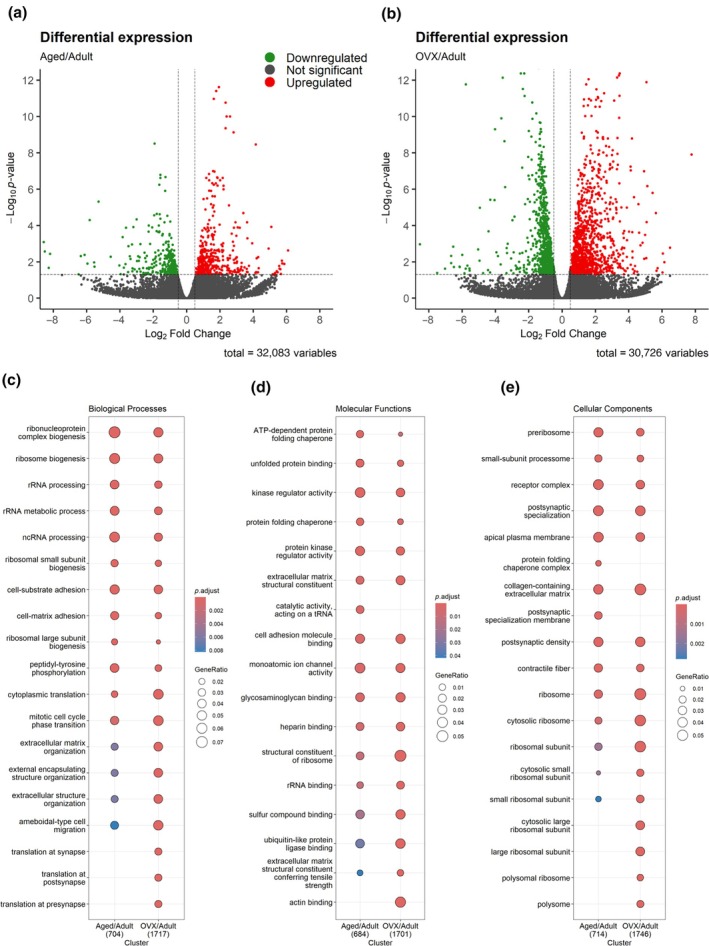
Volcano plots of differentially expressed genes (*p* ≤ 0.05 and Log_2_ Fold Change 0.5) in Aged versus Adult (a) and in OVX versus Adult (b). Comparative GO term enrichment analysis between Aged and Adult and OVX and Adult. Differentially expressed genes were submitted for Gene Ontology (GO) overrepresentation analysis using clusterprofiler in R. The top 10 overrepresented GO terms in the data set for biological processes (c), molecular functions (d) and cellular components (e). Representation of data in the estradiol deficient condition (Aged or OVX) relative to the control, Adult estradiol replete condition. Significant GO terms were accepted at *p*.adjust ≤0.05.

To identify satellite cell molecular factors that are similar or differ in two separate conditions of estradiol deficiency, ovariectomy and aging, RNAseq was completed comparing satellite cells from muscles of (1) Aged versus Adult and (2) Adult ovariectomized (Ovx) versus Adult ovary‐intact (Adult) female mice. In our two models of estradiol deficiency a total of 32,083 and 30,726 gene transcripts were identified in Aged/Adult and Ovx/Adult data sets, respectively. Further analysis revealed that 856 and 2040 genes were differentially expressed between Adult/Aged and Ovx/Adult satellite cells, respectively. Of the 856 differentially expressed genes between Adult and Aged, 439 are significantly upregulated and 417 are significantly downregulated with age (Figure 6a). Of the 2040 differentially expressed genes between satellite cells from Ovx and Adult‐ovary intact, 1116 are significantly upregulated and 924 are significantly downregulated with Ovx (Figure 6b).

In order to identify if these two models of estradiol deficiency have similar transcription patterns in satellite cells comparative GO term overrepresentation analysis was performed. The top 10 most overrepresented GO terms for biological processes, molecular functions and cellular components are shown in Figure 6c–e. Significant overrepresentation of GO terms (FDR <0.05) was present across all GO domains with significant overlap between both datasets. Overlap in processes and functions such as muscle cell proliferation/differentiation, cell cycle phase transition, growth factor binding/activity, and transcription/translation activity suggest that estradiol deficiency contributes to changes in satellite cell functions and/or depletion of the satellite cell pool with age in females. A few processes and cellular components were distinctly different between the two estradiol‐deficient conditions. While synaptic processes in satellite cells were affected by ovariectomy, they were not affected by aging suggesting that the presence of ovarian hormones is more important in Adult than Aged muscle (Figure 6c). Similarly, satellite cell ribosomal components were overall more affected by estradiol deficiency via ovariectomy than aging, despite being featured in both estradiol‐deficient conditions (Figure 6e).

Select overrepresented GO terms in both data sets are in Figure S1. GO biological processes consistently had similar terms for ribosome and rRNA processing, cell cycle and muscle proliferation, differentiation, and development in satellite cells from both estradiol‐deficient conditions. GO molecular functions had significant overlap for ribosome and rRNA binding, transcription and translation activity, growth factor binding, and activity and kinase activity between satellite cells from the two conditions. GO cellular components consistently had similar terms for ribosome and muscle sarcomere and associated structures. The high degree of similarity between the two groups suggests that estradiol deficiency accounts for a substantial portion of transcriptional changes in satellite cells with aging.

## 4 DISCUSSION

Age‐associated deficits in skeletal muscle including those in satellite cells can be driven by cell intrinsic or extrinsic factors that differ between adult and aged environments (Brack & Rando, 2007; Chakkalakal et al., 2012; Dumont et al., 2015). Estradiol is a particularly interesting extrinsic factor as levels fall naturally in females at menopause or ovarian senescence and our previous work has shown that Ovx of adult mice drastically reduces satellite cell number and function (Collins et al., 2019; Larson et al., 2022). Further, estradiol replacement in adult Ovx mice maintains the satellite cell pool (Collins et al., 2019; Larson et al., 2020, 2022). In the present study we tested the hypothesis that loss of estradiol with natural aging contributes to a depletion of the satellite cell pool in Aged female mice. To test this hypothesis, we first demonstrated that satellite cell number is ~48% lower in select hindlimb muscles (absolute number in the TA and EDL and per mg in the TA, EDL and Gas) of Aged compared to Adult female mice. In subsequent experiments, we manipulated the estradiol deficient environment of Aged satellite cells by supplementing ovarian senescent mice with estradiol, transplanted Aged and Adult satellite cells into an estradiol replete, Adult environment, or transplanted Adult satellite cells into either an Adult or Aged environment. Additionally, we isolated Aged and Adult satellite cells and cultured them in the same proliferation promoting environment and we isolated Adult satellite cells that had been transplanted into either Adult or Aged mice and cultured them. These results support our hypothesis by demonstrating that ovarian senescent mice supplemented with estradiol have greater satellite cell numbers in multiple hindlimb muscles and that the transplantation of satellite cells from Adult (estradiol replete) and Aged (estradiol deficient) mice into Adult recipients with circulating estradiol show similar engraftment, while Adult satellite cells transplanted into Aged recipients have worse satellite cell pool reconstitution capacity than those transplanted into an Adult environment. Further, these same satellite cells show similar proliferative and self‐renewal abilities when cultured in the same environment in vitro.

Aging is associated with a loss of skeletal muscle mass and strength (Messier et al., 2011) and in aged humans and mice anabolic resistance and reduced responsiveness to hypertrophic stimuli has been reported (Breen & Phillips, 2011; Hodson et al., 2019; van Dijk et al., 2017). Additionally, the loss of muscle mass and strength with age is accompanied by a significant loss of satellite cell number and function contributing to reduced capacity for muscle regeneration (Fry et al., 2015; Sousa‐Victor et al., 2022; Sousa‐Victor & Muñoz‐Cánoves, 2016). Many studies utilizing mouse models of aging have focused on males likely to avoid complications of sex/sex hormones (Arpke et al., 2021; Baumann et al., 2019; Gilmer et al., 2023; Phillips et al., 1993; Xie et al., 2021). This has left a dearth of research examining the age‐associated loss of satellite cells in female mice. Some reports have examined the loss of satellite cell number with aging in both sexes, highlighting that the loss of satellite cells is more pronounced in aged females than males (Day et al., 2010; Neal et al., 2012). However, these previous studies only examined EDL muscle and only quantified satellite cells on isolated myofibers. In the present study, we observed that satellite cell number is ~48% lower in select hindlimb muscles of Aged versus Adult female mice. These findings recapitulate what has been previously reported in male mice (Arpke et al., 2021; Day et al., 2010; Neal et al., 2012) and female (Day et al., 2010; Neal et al., 2012) and studies on men (Renault et al., 2002; Verdijk et al., 2014) but with the added robustness of analyzing satellite cell content in whole muscles. We previously showed that the muscles that are most depleted of satellite cells with age in males are those muscles most affected by lack of physical activity inherent in housing in conventional cages, that is, locomotory muscles (Arpke et al., 2021), and this result was also borne out in females, with the most significant decline seen in the TA, followed by the EDL and more modest effect sizes seen in Gas and Sol, for the most part not reaching statistical significance. These locomotory muscles represent a diverse composition of fiber types while Ovx does not alter fiber composition (Moran et al., 2007) and aging has not been reliably shown to alter fiber type in mice. These results therefore amplify the caveat that decline in satellite cell numbers in caged aged mice integrate both aging and sedentariness.

We hypothesized that the loss of satellite cell number observed in female mice is the result of a loss of estradiol due to ovarian senescence. In support of the hypothesis, ovarian senescent mice that received estradiol supplementation for 16 weeks had higher satellite cell number in the TA and Sol muscles compared to mice that received placebo treatment. These data provide compelling evidence that the loss of estradiol that occurs with ovarian senescence contributes to the depletion of the satellite cell pool in aging females. However, our reduced *n* size due to the loss of aged mice during the 16‐week treatment period is limiting. This finding is consistent with our previous reports that Adult Ovx mice treated with estradiol have a preserved satellite cell pool compared to Ovx mice treated with placebo (Collins et al., 2019). In the present study, we supplemented Aged mice with a dose of estradiol approximately 50% of what we have previously supplemented Adult mice with following Ovx due to previous observations of bladder issues in Aged mice (Greising et al., 2011).

In addition to the loss of satellite cell number with age, aging is associated with reduced satellite cell proliferation, self‐renewal and regenerative potential (Day et al., 2010; Sahinyan et al., 2023; Sousa‐Victor et al., 2022; Sousa‐Victor & Muñoz‐Cánoves, 2016). Currently it is unclear to what extent cell intrinsic age versus the aging environment is primarily responsible for impairments in maintenance of the satellite cell pool (Brack & Rando, 2007; Chakkalakal et al., 2012; Novak et al., 2021; Sousa‐Victor et al., 2022). To test our hypothesis that the loss of estradiol during aging is a critical environmental factor that is necessary for maintenance of the satellite cell pool in female mice, we transplanted 300 ZsGreen+ satellite cells isolated from Aged (estradiol deficient) and Adult (estradiol replete), female mice into Adult (estradiol replete), female recipients following irradiation and cardiotoxin injury. No differences were observed between Adult and Aged satellite cells' ability to engraft into Adult recipients, indicating that cell intrinsic age (i.e., Adult or Aged donor) has no impact on the ability of cells to engraft and proliferate in an estradiol‐replete environment. Colony‐forming assays performed on Adult and Aged donors satellite cells revealed no differences in self‐renewal or proliferative potential. These data agree with our previous findings in males (Arpke et al., 2021) as well as transplantation of female cells demonstrating poor engraftment of cells from Adult donors into an estrogen deficient environment (Ovx recipient) versus satellite cells implanted into an estrogen replete environment (Collins et al., 2019). Additionally, results from our second transplant strengthen our hypothesis by demonstrating that Adult satellite cells have worse engraftment into an Aged environment than an Adult environment. A potential limitation of these transplant experiments is the high built‐in variability, however our lab has consistently published results in which experimental conditions are significant (Arpke et al., 2021; Collins et al., 2019). Interestingly, when the transplanted Adult satellite cells were isolated from Aged and Adult recipients following transplantation and cultured, EdU incorporation was reduced in satellite cells from Aged recipients, however, no differences in clonogenicity were observed. Of note, the EdU incorporation is done in satellite cells cultured for only 30 h, whereas the colony‐forming assay takes 8 days. Therefore, these findings likely are due to the aged environment generating a deep but reversible quiescence, allowing normal clonogenicity. Further supporting these findings is our previous report demonstrating that estradiol signaling through ERα is essential in females for satellite cell proliferation and engraftment following transplantation (Collins et al., 2019; Larson et al., 2020, 2022). Together, these findings indicate that environmental factors, such as the presence or lack of sex hormones, may contribute to differences in satellite cell proliferation, self‐renewal and regenerative potential between Adult and Aged mice observed in previous research (Day et al., 2010; Sahinyan et al., 2023; Sousa‐Victor et al., 2022; Sousa‐Victor & Muñoz‐Cánoves, 2016).

We performed RNA‐Seq analysis on satellite cells isolated from 3 different groups, Adult (ovary‐intact), Ovx and Aged (ovarian senescent) in order to identify the transcriptional changes that occur with age that can be accounted for by the loss of ovarian hormones. We opted to compare both conditions of estradiol deficiency to the same control group so that each analysis compared just one factor; age factor in Aged versus Adult and ovarian factor in Ovx versus Adult (ovary‐intact). Of note, there were no differences in Esr1 transcripts between Adult and Aged mice but an increase in Ovx was observed (Adult = 2538.25 ± 825.75; Aged = 2577.25 ± 439.00; OVX = 4559.75 ± 302.75), which is consistent with PCR data from our previous reports on the whole muscle of Ovx mice (Baltgalvis et al., 2010). Our analysis found significant overlap in the transcriptional alterations that occur with Ovx and age, that is, the two conditions of estradiol deficiency. Specifically, GO biological processes terms related to cell cycle and muscle cell proliferation, differentiation and muscle development were featured consistently in both groups. GO molecular functions terms related to ribosome/ rRNA and RNA binding, growth factor binding/activity, transcription activity/ binding and various kinase activities were featured in both groups. GO cellular components terms for the ribosome and various aspects of skeletal muscle and associated contractile apparatus were featured throughout both groups. These results are in agreement with our previous findings in Adult, Aged and Ovx female mice in which the phosphoproteome of skeletal muscle was similarly altered in Aged and Ovx mice (Peyton et al., 2023), suggesting that the loss of estradiol, whether due to removal or senescence of the ovaries, induces transcription patterns in satellite cells that negatively impact the maintenance of the satellite cell pool.

In conclusion, our findings support the hypothesis that in female mice, estradiol deficiency is a defining characteristic of aging that contributes to the depletion of the satellite cell pool in Aged females. The loss of satellite cell number likely contributes to impaired muscle regeneration, impairing the recovery of strength and ultimately negatively impacting quality of life. Therefore, these findings highlight the vital role that estradiol plays in regulating muscle mass in females, and have implications for the treatment of women undergoing menopausal transition.

## AUTHOR CONTRIBUTIONS

BPS, AAL, MK and DAL conceived and designed research; BPS, AAL, SLM, AAS and MCE performed experiments; BPS analyzed data, interpreted results of experiments prepared figures and drafted the manuscript; BPS, MK, DAL, edited and revised the manuscript. All authors approved the final version of the manuscript.

## FUNDING INFORMATION

This study was funded by the National Institute of Health (NIH) grants R01‐AG062899 (DAL and MK). SLM and BPS were supported on T32‐AR007612. AAL was support on T32‐AG029796.

## CONFLICT OF INTEREST STATEMENT

None declared.

## Supporting information


Figure S1.

## Data Availability

The data that support the findings of this study are available in the NCBI SRA under the following ascension number (PRJNA1145450). All other data are available from the corresponding author upon reasonable request. Reviewer link to RNA‐Seq data: https://dataview.ncbi.nlm.nih.gov/object/PRJNA1145450?reviewer=f5m6aj8ol6dl6pqenvg6rd3e1b.
