# Supplementary material for: Estradiol deficiency as a consequence of aging contributes to the depletion of the satellite cell pool in female mice
Source: Aging Cell. 2024 Dec 6;24(4):e14441. doi: 10.1111/acel.14441 (PMC11984698; doi:10.1111/acel.14441)
Supplement: Supplementary file 1 — Figure S1. [file ACEL-24-e14441-s001.docx]

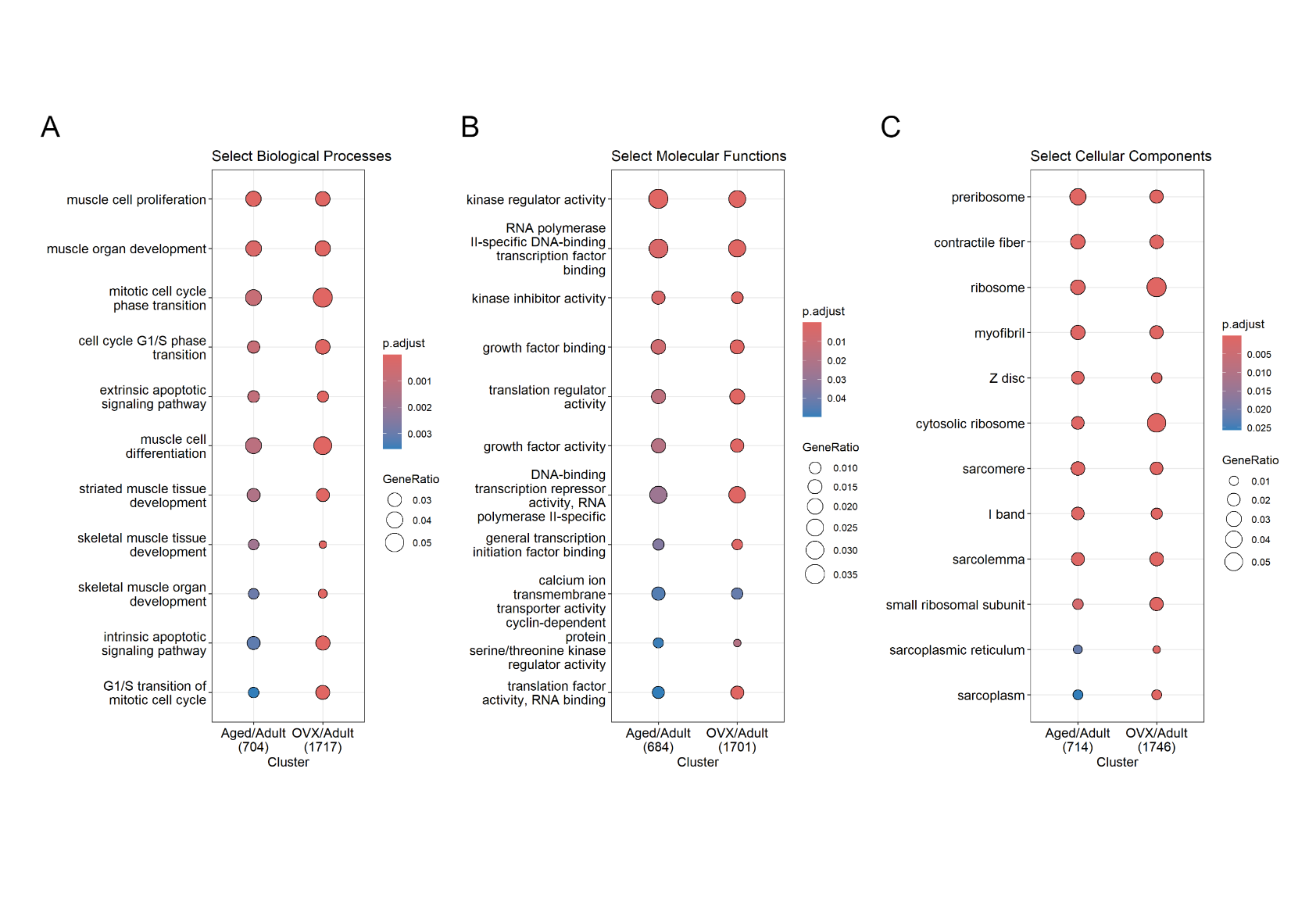


Supplemental Figure 1. A number of GO terms responsive to estradiol deficiency and/or have implications for satellite cell function were present in both conditions. Relevant overrepresented GO terms for biological processes (A), molecular functions (B) and cellular components (C). Representation of data in the estradiol deficient condition (Aged or OVX) relative to the control, Adult estradiol replete condition. Significant GO terms were accepted at *p*.adjust ≤ 0.05.


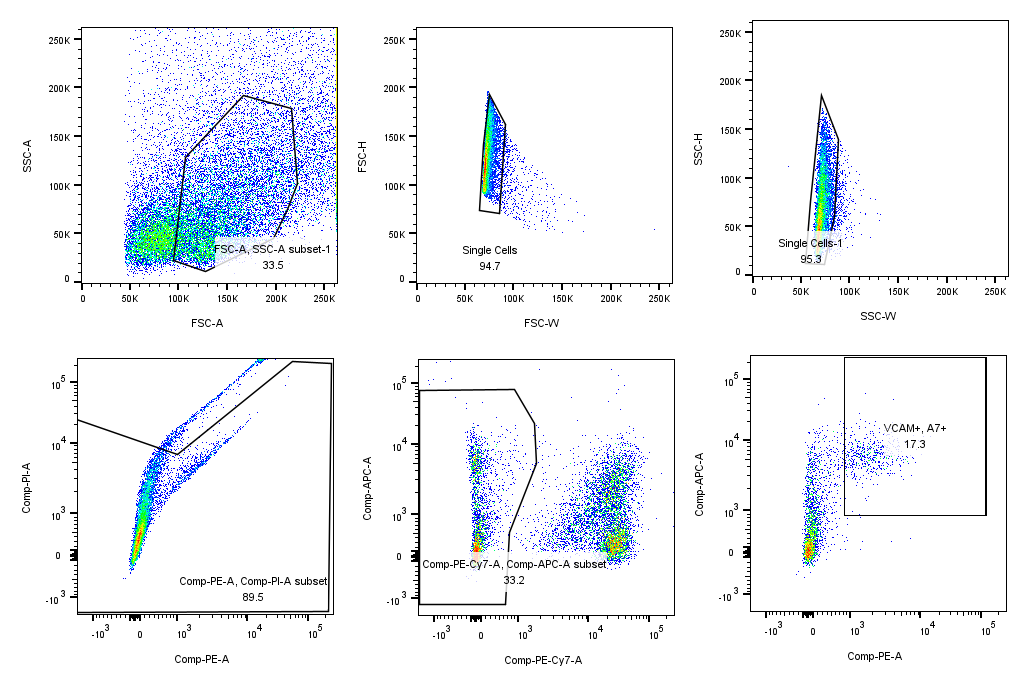


Supplementary Figure 2. Gating example of VCAM, Integrin α7 double positive cells.


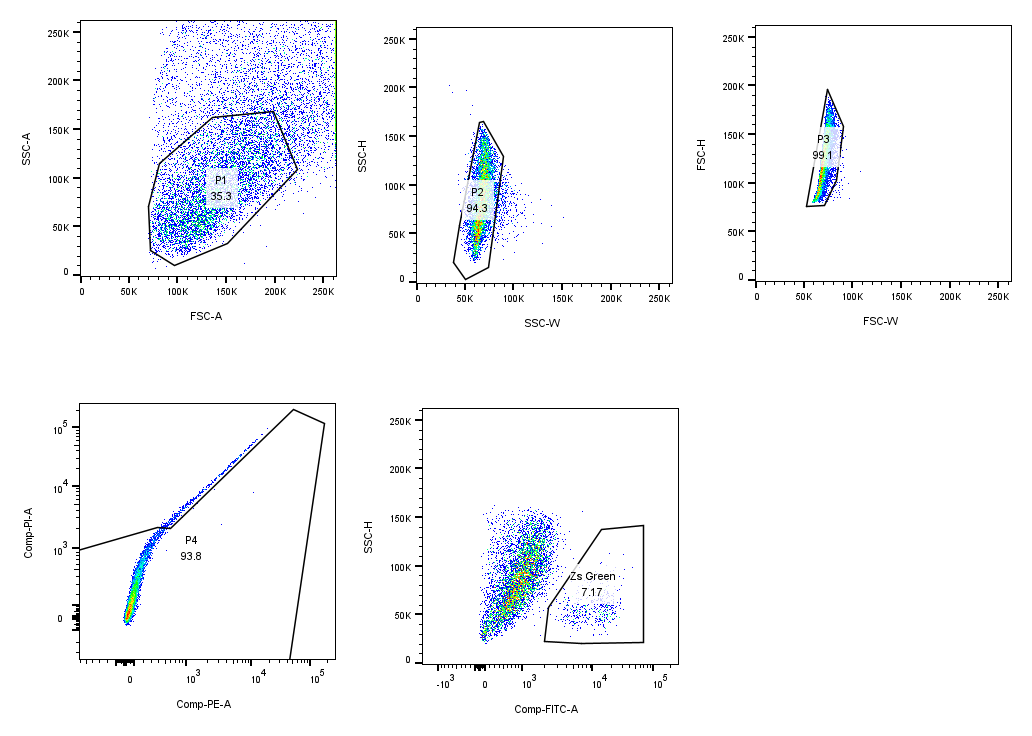


Supplementary Figure 3. Gating example of Pax7ZsGreen+ cells.
